# Distributional effects of macroeconomic shocks in real-time: A novel method applied to the COVID-19 crisis in Germany

**DOI:** 10.1007/s10888-021-09489-4

**Published:** 2021-09-20

**Authors:** Kerstin Bruckmeier, Andreas Peichl, Martin Popp, Jürgen Wiemers, Timo Wollmershäuser

**Affiliations:** 1 Institut für Arbeitsmarkt- und Berufsforschung (IAB) der Bundesagentur für Arbeit (BA), Regensburger Straße 104, 90478 Nürnberg, Germany; 2 ifo Zentrum für Makroökonomik und Befragungen, Poschingerstraße 5, 81679 München, Germany

**Keywords:** Income distribution, Inequality, Recession, COVID-19, Tax-benefit policies, Short-time work, Business survey, Labor demand, Microsimulation

## Abstract

The highly dynamic nature of the COVID-19 crisis poses an unprecedented challenge to policy makers around the world to take appropriate income-stabilizing countermeasures. To properly design such policy measures, it is important to quantify their effects in real-time. However, data on the relevant outcomes at the micro level is usually only available with considerable time lags. In this paper, we propose a novel method to assess the distributional consequences of macroeconomic shocks and policy responses in real-time and provide the first application to Germany in the context of the COVID-19 pandemic. Specifically, our approach combines different economic models estimated on firm- and household-level data: a VAR-model for output expectations, a structural labor demand model, and a tax-benefit microsimulation model. Our findings show that as of September 2020 the COVID-19 shock translates into a noticeable reduction in gross labor income across the entire income distribution. However, the tax benefit system and discretionary policy responses to the crisis act as important income stabilizers, since the effect on the distribution of disposable household incomes turns progressive: the bottom two deciles actually gain income, the middle deciles are hardly affected, and only the upper deciles lose income.

**Supplementary Information:**

The online version contains supplementary material available at 10.1007/s10888-021-09489-4.

## 1 Introduction

The SARS-CoV-2 virus pandemic confronts the world with a rapid spread of infections associated with COVID-19. Most governments around the world use interventions such as social distancing measures, school closures, curfews, and restrictions of business activity to slow down and contain the pandemic. While evidence suggests that these measures indeed

✉ Jürgen Wiemers
juergen.wiemers@iab.de

Extended author information available on the last page of the article.

reduce the number of infections, they also give rise to substantial economic costs with potential distributional consequences (Dorn et al. 2020). For policymakers it is important to assess these costs in real-time in order to design potential counteracting policy measures. However, data on the relevant outcomes is usually not available in real-time but only with considerable time lags.

In this paper, we propose a novel method to assess the short-term effects of macroeconomic shocks on the income distribution in real-time. We apply our method in the context of the COVID-19 pandemic in Germany and provide the first distributional analysis for this setting. To be precise, we investigate the short-term effects of a negative output shock on labor demand and on distributional outcomes accounting for policy responses to counteract the crisis. Germany is an interesting case study of a large, internationally integrated economy that was confronted with a sharp increase in SARS-CoV-2 infection numbers at the beginning of 2020 and responded with strong countermeasures. The lockdown measures taken at the end of March led to a sudden and severe collapse in economic activity. Since then, Germany has been fighting against an impending recession with massive short-time work programs for employees and financial aid for companies.

Our approach uses different data sources and economic models and can be updated regularly. In terms of methods, we develop and combine five ingredients in a novel way. First, we exploit information from a monthly panel survey of German firms to estimate output shocks at the industry-level in real-time using vector autoregressive (VAR) models.[1] Second, we use a structural labor demand model estimated on linked employer-employee data to measure the impact of output changes on employment (both at the extensive and intensive margin) of heterogeneous worker types. Third, we interact the estimated industry-level output shocks with output elasticities from the labor demand model to obtain fine-grained information on employment changes by industry and worker type due to the COVID-19 crisis. Conceptually, these effects cover both transitions from employment into short-time work or unemployment and transitions from unemployment into employment. Fourth, this is the first paper to use the maximum entropy principle to feed these predicted shocks to household micro data. Crucially, the maximum entropy principle allows us to relax extreme (and likely inadequate) assumptions on the distribution of hours reductions for workers. Fifth, we use a microsimulation model to assess the distributional consequences of both the containment measures as well as the policy responses in terms of income support for households. We analyze the distributional effects with respect to four different income concepts: gross labor income, net income of the working population, net income of the total population and disposable income of the total population accounting for discretionary non-employment responses to the pandemic. In order to show the dynamics of the crisis and its impact on our findings, we report results that are based on information collected at three different points in time: April, June and September 2020.

Our main findings can be summarized as follows. Based on the information from the monthly business survey, the pandemic is expected to lead to a significant decline in output in most industries in 2020. This output shock translates into a noticeable reduction in

[1] Note that the aim of our analysis is to quantify the distributional effects that the macroeconomic shock (on total output) resulting from the pandemic has on disposable incomes of households. The cause of this shock, i.e., whether it is due to, e.g., closure of firms because of non-pharmaceutical interventions to contain the spread of the virus, due to problems in the supply-chain (e.g., because of border controls), due to behavioral adjustments of consumers or any combination of these type of shocks (or due to some other cause) does not matter for our analysis. Therefore we use the firm sentiment indicators from the business survey as a reduced form estimate of the shock on total output in the economy (independent of the underlying cause).

both labor demand and gross labor income. All working households across the income distribution suffer from income losses, with the highest income losses experienced in the first decile. In contrast to the 2008/09 recession, which primarily affected the manufacturing sector (Bargain et al. 2012), the shutdown of economic activities in 2020 also severely impacted the service sector, in particular the hotel and restaurant industries and the travel industry. The tax benefit system acts as an important automatic stabilizer as expected losses in disposable income are significantly reduced for affected working households. It also changes the structure of income losses across the income distribution. Lowest net income losses are experienced in the middle part of the income distribution and highest in the first, eighth, ninth and tenth decile groups. As a disproportionately large number of inactive people, e.g., recipients of means-tested benefits, are represented in lower deciles, the average relative income losses in the lower decile groups become smaller when including non-employed households in the analysis. Finally, accounting for the discretionary non-employment policy measures enacted as a response to the recession, the effect on the total disposable income distribution turns progressive. Specifically, the first two decile groups actually gain income. In contrast, the middle part of the distribution is hardly affected while the upper deciles still lose income. This strong redistributive effect of the discretionary non-employment measures is mainly driven by the child bonus, a progressive benefit paid to support families during the crisis.

Note that this approach comes with some limitations. The distributional effects are based only on first-round labor market effects with assumed constant wages. Given that we are interested in the short-term effects of the crisis and the long-term effects of the crisis are uncertain, this does not seem to be a very restrictive assumption. Moreover, our estimated employment changes rely purely on aggregate shocks per industry and worker type and not on individual real-time data (e.g., collected via surveys). Therefore, our approach cannot capture differentiated income shocks on specific socio-economic subgroups, e.g., migrants or single parents.

Our results are broadly consistent with other studies on the distributional effects of the pandemic for Germany. Almeida et al. (2021) make use of EUROMOD and EU-SILC data and model an aggregated employment shock based on forecasts of the European Commission, which is combined with the microdata by reweighting techniques. They find a strong stabilizing effect of the tax-benefit-system in Germany, a slight decrease in income inequality and a small increase in the poverty rate. The main difference to our analysis besides the modelling of the macroeconomic shock is that they analyze redistribution based on the tax-benefit-system of 2019, which should reduce the redistributive effect, as discretionary social policies introduced after the crisis are not considered. Beznoska et al. (2020) use the same microdata for the microsimulation as our analysis (SOEP) but model the income shock based on individual data from a survey conducted in August 2020 on the economic effects of the crisis. Shocks are integrated into the microsimulation model data by individual statistical matching. They simulate a larger negative income effect on gross incomes, but a comparable effect on disposable incomes of the total population. Furthermore, they also find a slightly positive income effect in the first two income deciles and an increasing negative effect up to the tenth income decile, also suggesting a progressive total effect.

These findings for Germany are in line with international evidence, e.g., Brewer and Tasseva (2020) and Bronka et al. (2020) for the UK and O'Donoghue et al. (2020) for Ireland. These papers suggest similar basic patterns and mechanisms behind the distributional effects, which are a) a decline of the overall negative effects on the income distribution during the crisis, b) the importance of short-time work schemes as the main insurance mechanism, and c) a progressive total income effect with income gains in the lower tail of the income distribution and a reduction in the Gini coefficient and the poverty rate due to non-employment policy measures. Our results also emphasize the importance of (access to) unemployment insurance and the short-time work program as an automatic stabilizer (Bargain et al. 2012; Dolls et al. 2012). Further studies show that although political interventions in all European countries have helped cushioning the income losses caused by the crisis, the extent of the cushioning effect varies greatly (Almeida et al. 2021; Figari and Fiori 2020).

The tools and methods developed here are also applicable to many other countries in which labor market and distributional microdata is not available in real-time. For example, comparable business surveys exist in a large number of economies, including the US and EU (e.g., through the EU Economic Sentiment Indicator (ESI) surveys). Linked employer-employee data to estimate labor demand elasticities are also widely available (Abowd and Kramarz 1999). Alternatively, results from meta-studies could be used (Lichter et al. 2015). Finally, microsimulation models (such as TAXSIM for the US or EUROMOD in the EU) exist in many countries.

## 2 Methodology and data

In this paper, we develop and integrate different models to assess the distributional consequences of the COVID-19 pandemic in real-time. In the following section, we describe these ingredients, their underlying data and intermediate results used for the distributional analysis: the monthly business survey to estimate output shocks at the industry-level in real-time (Section 2.1), the labor demand model to obtain fine-grained information on employment changes by industry and worker groups (Section 2.2), and the microsimulation model to evaluate the distributional consequences (Section 2.3) of both the containment measures as well as the implemented policy responses (Section 2.4).

### 2.1 Estimating output shocks

Output of firms is typically measured as gross value added at constant prices (GVA). For our analysis of the distributional consequences of the COVID-19 crisis, output data at the 2-digit NACE industry level is required. However, at this level only annual data is published by the Federal Statistical Office of Germany, and only with a considerable lag of about 20 months. Thus, at the beginning of September 2020, data for the year 2018 was released. To obtain real-time estimates of the impact of the COVID-19 crisis on firm-level output in 2020, we make use of information from the monthly ifo Business Survey (iBS), which collects firm-level perceptions of business activity.

**Data** The iBS is a monthly survey covering roughly 9,000 responses of German firms in manufacturing, construction, trade and services (Becker and Wohlrabe 2008; Sauer and Wohlrabe 2020). The firms included in the survey represent roughly 74 percent of the total German economic output and cover industries that were more than proportionally affected

**Table 1** Elasticity of GVA with respect to ifo business situation

| $i$ | C | F | G | H | I | J | L | M,N | R,S,T |
|---|---|---|---|---|---|---|---|---|---|
| $c_0$ | 0.16 | – 0.13 | 0.39** | 0.25 | – 0.50 | 1.14*** | 0.26** | 0.22 | – 0.24 |
| $c_1$ | 0.26*** | 0.14*** | 0.12*** | 0.13*** | 0.69*** | 0.11*** | 0.01 | 0.25*** | 0.30 |
| Obs. | 117 | 117 | 117 | 61 | 61 | 61 | 61 | 61 | 61 |
| $R^2$ | 0.59 | 0.05 | 0.16 | 0.42 | 0.74 | 0.18 | 0.00 | 0.59 | 0.36 |

For NACE sections C, F, and G the sample period runs from Q1 1991 to Q2 2020. Since the ifo Institute only started in 2005 to integrate firms from the service sector to the iBS, all regressions using the business situation in section H and the following have a lower number of observations. For section R,S,T quarterly GVA is only available for the aggregate and not for each NACE section individually. Since industry T is not covered by the iBS, GVA in industry R,S,T is regressed on the business situation in industry R,S only. C = Manufacturing. F = Construction. G = Wholesale and Retail Trade, Repair of Motor Vehicles and Motorcycles. H = Transportation and Storage. GVA = Gross Value Added at Constant Prices. I = Accommodation and Food Service Activities. iBS = ifo Business Survey. J = Information and Communication. L = Real Estate Activities. M = Professional, Scientific and Technical Activities. N = Administrative and Support Service Activities. NACE = Statistical Classification of Economic Activities in the European Community. Obs. = Observations. R = Arts, Entertainment and Recreation. S = Other Service Activities. T = Activities of Households as Employers, Undifferentiated Goods- and Services-Producing Activities of Households for Own Use. * = p<0.10. ** = p<0.05. *** = p<0.01. Sources: iBS + Federal Statistical Office of Germany, 1991–2020

by the COVID-19 crisis.[2] According to statistics of the Federal Employment Agency around 87 percent of all short-time workers were employed in these sectors in April 2020.

Each month firms are asked to give their assessments of the current business situation, which they can characterize as “good”, “satisfactorily” or “poor”. The replies are weighted with the size of the firms, aggregated to industry levels, and released at the end of the month in which the survey was conducted as a balance statistic. For each industry level, this balance statistic is defined as the difference of the percentages of the responses “good” and “poor”. For the aggregate economy and most industries, changes in the business situation are highly correlated with changes in GVA (Lehmann 2020). To show this, we run the following regressions for each industry $i$

$$\Delta \ln(Y_{i,t}) = c_0 + c_1 \Delta BS_{i,t} + \varepsilon_{i,t}, \tag{1}$$

where $BS_{i,t}$ denotes the business situation and $Y_{i,t}$ GVA in quarter $t$. Industries are aggregated to the level of economic sections (i.e., the 1-digit industry level), for which the Federal Statistical Office of Germany publishes quarterly data shortly after the end of the quarter. The results of the regressions are summarized in Table 1. Most of the elasticities of GVA with respect to the ifo business situation ($c_1$) are positive and statistically significant, implying that changes in the business situation are sufficiently precise and timely indicators for current output changes.

[2]The survey excludes public services summarized by NACE sections O, P and Q (public administration, defence, compulsory social security, education, human health and social work activities), as well as some small industries which play virtually no role for economic fluctuations (A: Agriculture, forestry and fishing; B: Mining and quarrying; D: Electricity, gas, steam and air conditioning supply; E: Water supply, sewerage, waste management and remediation activities; K: Financial and insurance activities; T: Activities of households as employers, undifferentiated goods- and services-producing activities of private households for own use; U: Activities of extraterritorial organizations and bodies). Public services account for 18.2 percent of 2017 total gross value added, the other excluded industries for 8.1 percent.

**Output shocks** We define output shocks as unexpected changes in output forecasts for the year 2020. For the estimation of these shocks, we are additionally exploiting the firms' expectations with regard to the business development in the next six months. In the iBS, survey participants can characterize these expectations as "more favorable", "unchanged" or "more unfavorable". As with the business situation, the replies are summarized to a balance statistic. At the firm level, these business expectations are forecasts of a firm's future business situation. At the industry level, they are a natural candidate for a leading indicator of the business situation. To show this, we calculate cross-correlation coefficients $r$ for the two stationary series, which are defined as the correlation between the ifo business situation $BS$ in industry $i$ at time $t$ and the ifo business expectations $BE$ in the same industry at time $t-h$. Table 2 shows that for most industries (for simplicity aggregated again to the 1-digit level) business expectations are leading the business situation by several months. Moreover, the respective correlation is high enough such that expectations can be used to predict future business situations.

The most straightforward approach to exploit the information contained in the business expectations for forecasting the business situation is to set up a bivariate vector autoregressive (VAR) model. For each industry $i$ at the 2-digit level, we estimate the following VAR models

$$y_{i,t} = c_i + \sum_{h=1}^{p} A_h y_{i,t-h} + \varepsilon_{i,t}, \tag{2}$$

over the period ranging from $T_0$ (which is January 2005 for industries in the service sector and January 1991 for all other industries) to $T_1 \in$ {February 2020, April 2020, June 2020, September 2020}, where $y_{i,t}$ is a $2 \times 1$ vector containing the business situation $BS_{i,t}$ and the business expectations $BE_{i,t}$, $c_i$ is a $2 \times 1$ vector of constants, $A_h$ is for each lag $h$ a $2 \times 2$ matrix of estimated coefficients, and $\varepsilon_{i,t}$ are the white-noise innovation processes. All models are estimated separately for each industry $i$ using OLS with the same lag length $p = 12$.

The predictions are made for the months $T_1 + 1$ until the end of 2020, such that for each industry $i$ an expected average business situation for 2020 conditional on the information from the iBS at time $T_1$ can be calculated. In a next step for each $T_1$ the expected change of the business situation is transformed into expected percent changes of GVA in industry $i$ using the estimated elasticities $c_1$ shown in Table 1. As the elasticities are only available

**Table 2** Cross-correlation between ifo business situation and expectations

| $i$ | C | F | G | H | I | J | L | M,N | R,S |
|---|---|---|---|---|---|---|---|---|---|
| $h$ | 6 | 1 | 3 | 6 | 3 | 8 | 3 | 7 | 0 |
| $r_{BS,BE}(h)$ | 0.69 | 0.87 | 0.74 | 0.70 | 0.59 | 0.42 | 0.65 | 0.55 | 0.28 |

Cross-correlations are calculated using monthly data from 1991 (or 2005 when iBS data from sections H and the following is used) to 2019. The second row shows the lead $h$ of the ifo business expectations that maximizes the correlation between $BS$ and $BE$. The third row shows the related correlation coefficient. BE = ifo Business Expectations. BS = ifo Business Situation. C = Manufacturing. F = Construction. G = Wholesale and Retail Trade, Repair of Motor Vehicles and Motorcycles. H = Transportation and Storage. I = Accommodation and Food Service Activities. iBS = ifo Business Survey. J = Information and Communication. L = Real Estate Activities. M = Professional, Scientific and Technical Activities. N = Administrative and Support Service Activities. R = Arts, Entertainment and Recreation. S = Other Service Activities. Source: iBS, 1991–2019

for economic sections (i.e., the 1-digit industry level), we applied the same section-specific elasticity to all 2-digit industries of the corresponding section.

The forecasts made conditional on the information available up to February 2020 serve as reference for the calculation of the output shock as this month featured the last survey round prior to the outbreak of the COVID-19 crisis in Germany. In March and April, the assessments of the current business situation and the expectations with regard to the business development in the next six months dropped significantly. Given the information available at the peak of the crisis in April 2020 we predict the level of output in the surveyed part of the economy for 2020 to decline by 8.8 percent compared to the February forecast. In the following months business expectations improved much more quickly than predicted by the VAR model that was estimated using data up to April. Thus, it became more and more evident that the recovery of the German economy follows a V-shape instead of a more persistent U-shape, which was common in previous recessions. Accordingly, when iBS data of June and September were included into the VAR model, GVA forecasts for most industries were revised upwards, and the output shock for the total economy in 2020 was reduced to 6.5 percent in June and to 5.3 percent in September. The largest output shock between February and September 2020 was estimated for accommodation (− 43.7 percent) and food and beverage service activities (− 49.9 percent), the smallest for real estate and IT service activities (− 0.1 and − 0.7 percent, respectively).[3]

## 2.2 Estimating labor demand effects

Next, we seek to identify the effect of macroeconomic shocks on employment of heterogeneous groups of workers. To this end, we link our estimated output shocks with empirical estimates of the impact of output changes on the demand for labor. To do so, we first estimate output elasticities using a structural labor demand model.

**Labor demand model** We apply the dual approach and derive output elasticities of labor demand from a cost-minimization model (Hamermesh 1993). Accordingly, we assume establishments to select factor demands as to minimize cost given an exogenous level of production. We estimate parameters of a Translog cost function from Christensen et al. (1973), which is a logarithmic second-order Taylor approximation to an arbitrary twice-differentiable cost function. Importantly, the Translog cost function, unlike a Cobb-Douglas or CES cost function, does not impose any structure on the substitution possibilities between different input factors. We make use of the non-homothetic version of the Translog cost function from Berndt and Khaled (1979) and thereby allow cost-minimizing input ratios to vary with the level of production. As our goal is to model short-term demand responses to an unanticipated output shock, we assume that the capital stock is fixed and, hence, separable from the $M$ remaining labor inputs $H^m$ (measured in terms of hours). We follow Diewert and Wales (1987) and model technological progress as a quasi-fixed input factor for time $t$.

[3] Note that our results are robust to the choice of the lag length of the VAR model. If we set $p = 4$, the output shock in the surveyed part of the economy for 2020 conditional on information in April declines to − 8.9 percent from − 8.8 percent. The corresponding values for June and September increase to − 6.3 and − 4.8 percent, from − 6.5 and − 5.3 percent.

Our short-term, non-homothetic and multi-factor Translog cost function reads

$$\ln C_{j,t} = \beta_0 + \sum_{m=1}^{M} \beta_m \cdot \ln w_{j,t}^m + \frac{1}{2} \cdot \sum_{m=1}^{M} \sum_{n=1}^{M} \beta_{mn} \cdot \ln w_{j,t}^m \cdot \ln w_{j,t}^n$$
$$+ \beta_Y \cdot \ln Y_{j,t} + \frac{1}{2} \cdot \beta_{YY} \cdot \ln Y_{j,t}^2 + \sum_{m=1}^{M} \beta_{mY} \cdot \ln w_{j,t}^m \cdot \ln Y_{j,t}$$
$$+ \gamma \cdot t + \frac{1}{2} \cdot \gamma_t \cdot t^2 + \sum_{m=0}^{M} \gamma_m \cdot \ln w_{j,t}^m \cdot t + \sum_{m=0}^{M} \gamma_{mY} \cdot \ln Y_{j,t} \cdot t \quad (3)$$

where $C$ and $Y$ denote establishment-specific cost and revenues and $w^m$ is the hourly wage rate for input factor $m$.[4] We use panel subscripts $j$ and $t$ to label establishments and years. By default, we impose the symmetry condition, $\beta_{mn} = \beta_{nm}$, on the parameters and assume that the cost function reflects linear homogeneity in input prices: $\sum_{m=1}^{M} \beta_m = 1$ and $\sum_{m=1}^{M} \beta_{mn} = \sum_{n=1}^{M} \beta_{mn} = \sum_{m=1}^{M} \beta_{mY} = \sum_{m=1}^{M} \gamma_m = 0$. We apply Shephard's (1953) Lemma to Eq. 3 and thus derive a system of M cost share equations $s^m$, $m = 1, \ldots, M$:

$$s_{j,t}^m = \frac{\partial \ln C_{jt}}{\partial \ln w_{j,t}^m} = \beta_m + \sum_{n=1}^{M} \beta_{mn} \cdot \ln w_{j,t}^n + \beta_{mY} \cdot \ln Y_{j,t} + \gamma_m \cdot t \quad (4)$$

To counteract bias from unobserved time-constant heterogeneity, we account for establishment fixed effects by within-transforming Eqs. 3 and 4. In addition, we implement year fixed effects and add a vector of random error terms with zero mean and a constant covariance matrix. However, the fact that all cost shares always sum up to one renders our error term covariance matrix singular and non-diagonal. We therefore follow standard practice and drop the last cost share equation $s^M$ while normalizing all input prices by $w^M$. Specifically, we apply an iterative SUR estimation to ensure that our estimates are invariant to the choice of cost share equation to be eliminated. We estimate our resulting system of a cost equation and $M - 1$ cost share equations using Zellner's (1962) Seemingly Unrelated Regression (SUR).

To arrive at output elasticities of labor demand, we plug our SUR estimates, realized values, as well as fitted cost (shares) into the formula for output elasticities of labor demand derived from the Translog cost function (Bellmann et al. 1999):

$$\hat{\eta}_{j,t}^m = \left[ \hat{\beta}_{mY} \cdot \frac{\hat{C}_{j,t}}{w_{jt}^m} + \hat{C}_{jt} \cdot \frac{\hat{s}_{j,t}^m}{w_{j,t}^m} \cdot \left( \hat{\beta}_Y + \hat{\beta}_{YY} \cdot \ln Y_{j,t} + \sum_{n=1}^{M} \hat{\beta}_{nY} \cdot \ln w_{j,t}^n \right) \right] / H_{j,t}^m \quad (5)$$

As values vary across observations, we evaluate our elasticities $\hat{\eta}^m$ at sample means of reported and fitted values.

[4]Homotheticity implies that input ratios are independent of the level of output (Berndt 1991). The Translog cost function is homothetic in case $\beta_{mY} = 0$ applies for each input $m$. As a special case of homotheticity, the cost function is homogeneous of a constant degree in inputs for $\beta_{YY} = 0$. In such a case, the degree of homogeneity is $\frac{1}{\beta_Y}$. If further holds that $\beta_Y = 1$, the cost function reflects constant returns to scale.

**Data** We estimate our cost-minimization model on the Linked Employer-Employee Dataset (LIAB) from the German Institute for Employment Research (IAB) for the years 1999–2016. The LIAB dataset combines survey information on employers from the IAB Establishment Panel with administrative data on their respective workers from the Integrated Employment Biographies (IEB).

Among other sources, the IEB collects all employment notifications from workers obliged to pay social security contributions in Germany (Müller and Wolter 2020).[5] The IEB includes a large set of worker and job characteristics, such as information on contract type, skill, age, industry or daily gross wages.[6] We use the two-step imputation technique from Card et al. (2013) to impute right-censored wages above the upper-earnings limit on social security contributions.[7] We multiply imputed gross wages with the social security contribution rates of employers to obtain a holistic measure of daily labor cost per employment spell. For the years 2010–2014, we are able to merge the IEB with hours information from the German Statutory Accident Insurance. We apply the heuristic from Dustmann et al. (2020) and harmonize the hours information to depict contractual hours plus overtime. We impute the hours information for the remaining years and divide daily labor cost by daily contractual hours (incl. overtime) to arrive at our final measure of hourly labor cost.[8] For each establishment-year combination and input factor, we compute the sum of daily contractual hours and average hourly gross wages on 30 June of the respective year and link both variables to the IAB Establishment Panel. In doing so, our cost-minimization model captures variation along both the extensive margin (workers) and the intensive margin (hours).

The IAB Establishment Panel is an annual survey of about 15,000 German establishments (Ellguth et al. 2014). The term "establishment" describes a separate production unit with at least one worker subject to social security payments. The random sample includes strata for 10 size classes, 16 industries, and 16 federal states. In particular, we harness information on revenues from the IAB Establishment Panel. To ensure sufficient substitution possibilities, we drop establishments with less than 10 workers. Moreover, we exclude establishments operating in industries that are not covered by the iBS survey, including the public service sector (see Section 2.1).[9] As output shocks may translate into employment

[5] The data cover about 80 percent of the German workforce. Self-employed persons, civil servants and family workers are exempt from social security contributions.

[6] Industry codes refer to the German Classification of Economic Activities 2008 (WZ 2008) whose first four digits coincide with the NACE Rev. 2 definition. For the years 1999–2007, we impute industry affiliations by applying the heuristic from Eberle et al. (2011) to industry codes for the Classifications of Economic Activities 1993 and 2003.

[7] Card et al. (2013) propose a two-step procedure for the imputation of wages. In a first step, fitted wages from a Tobit regression are used to calculate mean wages per establishment (excluding the observation at hand). In a second step, a further Tobit regression with this variable as an additional regressor delivers final imputations. Specifically, we regress log daily wages of regular full-time workers on age, (square of) log establishment size, share of low-skilled and high-skilled workers within the establishment, share of censored observations excluding the observation at hand as well as dummies for German nationality, location in East Germany, one-person establishments, and establishments with more than ten full-time employees. Separate Tobit models are estimated for each interaction of year (18 waves), gender (2 groups) and education (3 groups). Our final distributions of uncensored daily wages are similar to those obtained from survey-based SOEP information, thus corroborating the plausibility of the imputation procedure.

[8] Specifically, for each combination of contract type (7 groups), gender (2 groups) and education (3 groups), we regress daily hours on a set of individual- and establishment-level covariates and use the fitted models to impute missing information on hours for the years 1999–2009 and 2015–2016.

[9] In addition, our assumption of cost-minimizing firms is unlikely to hold for public agents who may act in a budget-maximizing manner. In fact, contrary to production theory, estimated output elasticities of labor demand turn out to be negative for public services.

effects in unequal fashion, we run our cost-minimization model on four distinct sectors. Our final sample refers to 94,334 establishment-year observations from the following sectors: 40,811 in manufacturing (NACE: C), 10,223 in construction (NACE: F), 27,551 in trade and traffic (NACE: G-J), and 15,749 in private services (NACE: L-N, R, S).

**Worker heterogeneity** We are interested in the impact of output shocks on the demand for fine-grained groups of workers. The rich set of variables in our administrative data enables us to differentiate between $M = 12$ labor inputs per sector. We distinguish between two contract types, two skill levels and three age groups.[10] We divide workers into two groups based on their employment contract: a standard group of regular full-time workers and non-standard worker category comprising regular part-time workers, marginal employment, apprentices, workers in partial retirement and other employment types. In contrast to unskilled workers, skilled workers have passed vocational training and/or hold a university degree. Regarding age, we differentiate between young (15-34 years), middle-age (35-49 years) and old workers (at least 50 years).

**Output elasticities** Table 3 depicts the matrix of estimated output elasticities of labor demand. Production theory implies that a higher (lower) level of production leads to a higher (lower) demand for labor. In line with theory, our estimated output elasticities of labor demand uniformly show a positive sign across all input-sector pairs. The majority of estimates lies in the range from 0.4 to 1.0 which is well in line with the previous literature.[11] In most cases, non-standard unskilled workers feature higher elasticities than standard unskilled workers. Moreover, output elasticities generally decrease with age, i.e., older workers are exposed to a lower risk of being dismissed when production declines. Furthermore, elasticities turn out to be especially high in the manufacturing sector. We use Bootstrapped standard errors based on 25 replications. Apart from few exceptions in the construction sector, the elasticities are significantly different from zero at 1 percent levels. The only exceptions are old and unskilled construction workers with a standard employment contract (p=0.07) as well as unskilled middle-age (p=0.06) and unskilled old construction workers in non-standard employment (p=0.31).

**Labor demand effects** In a next step, we quantify the short-term decline in labor demand by input factor and industry due to COVID-19. Specifically, we are interested in the relative change in demanded hours for 2020 expected in either April, June or September 2020 over demanded hours expected in February 2020 when the pandemic had not yet materialized in Germany. To this end, we interact output shocks for 2020 by 2-digit NACE industries (see Section 2.1) with our matrix of estimated output elasticities of labor demand (see Table 3).

[10] In the standard implementation of a cost-minimization model, establishment-year observations with zero employment in at least one of the input categories would drop out of the estimation sample for lack of observed wage information. To avoid losing a large number of observations, we impute missing wage information by yearly average wages for the relevant input-sector pair. To ensure sufficient statistical power for the identification of output elasticities per worker group, we do not go beyond using 12 labor inputs although our later distributional analysis would benefit from an even higher disaggregation (such as for gender, occupation or nationality).

[11] Bellmann et al. (1999) differentiate between white- and blue-collar workers and three skill groups and find output elasticities between 0.6 and 0.8 for Germany. Using a similar input scheme, Bargain et al. (2012) find an average output elasticity of 0.69.

**Table 3** Output elasticities of labor demand

| Input | | Sector | Manu-facturing | Con-struction | Trade and traffic | Private services |
|---|---|---|---|---|---|---|
| Standard | Unskilled | Young | 0.93 | 0.86 | 0.57 | 0.75 |
| | | Middle-Age | 0.58 | 0.36 | 0.34 | 0.58 |
| | | Old | 0.50 | 0.24 | 0.27 | 0.53 |
| | Skilled | Young | 1.08 | 0.71 | 0.68 | 0.70 |
| | | Middle-Age | 0.87 | 0.57 | 0.54 | 0.63 |
| | | Old | 0.97 | 0.51 | 0.47 | 0.60 |
| Non-Standard | Unskilled | Young | 1.10 | 0.83 | 0.91 | 0.74 |
| | | Middle-Age | 0.72 | 1.71 | 0.78 | 0.45 |
| | | Old | 0.46 | 0.33 | 0.45 | 0.54 |
| | Skilled | Young | 0.90 | 0.54 | 0.78 | 0.64 |
| | | Middle-Age | 0.74 | 0.72 | 0.72 | 0.71 |
| | | Old | 0.48 | 0.50 | 0.59 | 0.57 |
| Observations | | | 40,811 | 10,223 | 27,511 | 15,749 |

The table illustrates estimated output elasticities of labor demand by input factor and sector. We derive the elasticities from a non-homethetic Translog cost function using Shephard's Lemma and evaluate the formulas at estimated SUR coefficients, sample means and fitted values. Standard employment refers to full-time workers with a regular contract whereas non-standard employment includes regular part-time workers, marginal employment, apprentices, workers in partial retirement and other employment categories. Skilled workers have completed vocational training and/or hold a university degree. Workers are divided into three age groups: young (15–34 years), middle-age (35–49 years) and old (at least 50 years). We run our cost-minimzation model on four different sectors: manufacturing (NACE: C), construction (NACE: F), trade and traffic (NACE: G-J) as well as private services (NACE: L-N, R, S). LIAB = Linked Employer-Employer Dataset from IAB. SUR = Seemingly Unrelated Regression. NACE = Statistical Classification of Economic Activities in the European Community. Source: LIAB, 1999–2016

As a result, we are able to provide a fine-grained picture on COVID-19 labor demand responses for 648 different input-by-industry cells (12 inputs × 54 industries).[12]

Given the information available in April 2020, labor demand in the overall economy is predicted to decrease by 4.9 percent due to the pandemic. When instead using the more recent output information from June and September 2020, we arrive at an overall reduction by 4.0 and 3.5 percent. Figure 1 illustrates the labor demand effects for 2-digit industries (in terms of weighted averages of the 12 underlying labor inputs), separately by information as of April, June and September 2020. We observe a reduction in labor demand throughout the German economy. The magnitude of these negative effects shows unseen levels since World War II, thus pointing to the possibility of considerable fiscal and distributional consequences from the COVID-19 pandemic. As of the latest available information from September 2020, the food and beverage serving activities (− 31.9 percent) as well as the accommodation sector (− 28.0 percent) are most severely affected. We further report substantial labor demand reductions for manufacturing of transport equipment (− 13.5 percent), manufacturing of wearing apparel (− 12.1 percent), travel agencies and tour operators (− 11.8 percent) and manufacturing of beverages (− 9.4 percent). Among others, we find comparably small contractions of around 1 percent for manufacturing of pharmaceuticals, telecommunications,

[12] Our differentiation goes far beyond that of Bargain et al. (2012) who model labor demand responses to the financial crisis in 2008/09 for 60 cells (12 inputs × 5 industries).

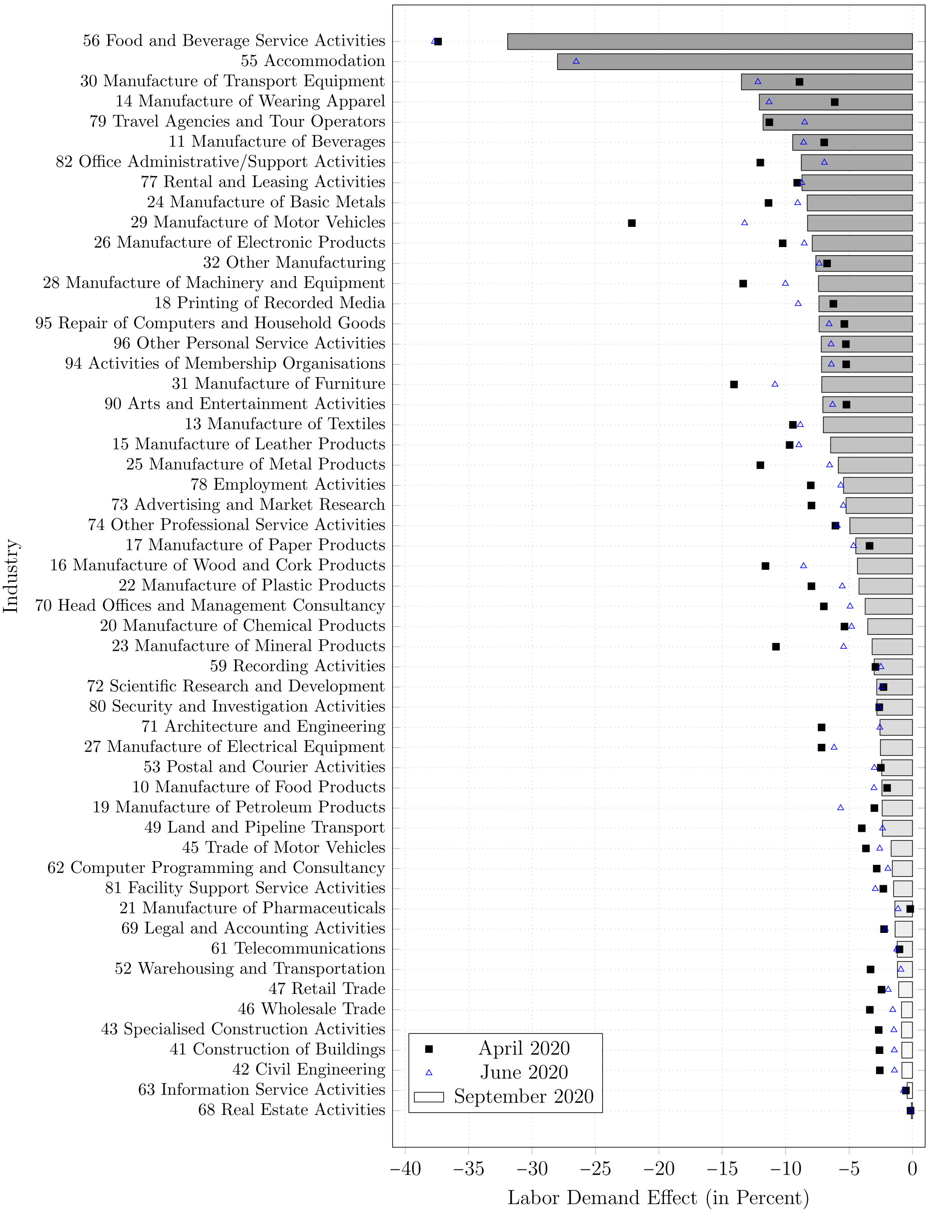

**Fig. 1** Labor demand effects by 2-digit industry. The figure displays the estimated impact of the COVID-19 pandemic on labor demand by 2-digit NACE industries. Specifically, for information as of April, June and September 2020, we interact predicted output shocks for 2020 by 54 2-digit NACE industries with our matrix of estimated output elasticities of labor demand (Table 3) to obtain relative labor demand effects for 648 input-by-industry cells. For each industry, we report an employment-weighted average of labor demand shocks over the twelve underlying labor inputs. The bars refer to the most recent information as of September 2020. In contrast, black squares and blue triangles describe labor demand effects based on information from April 2020 and June 2020, respectively. For reasons of space, the figure does not capture the year-on-year reduction in labor demand in the accommodation sector of − 57.1 percent as of April 2020. NACE = Statistical Classification of Economic Activities in the European Community. Sources: LIAB, 1999–2016 + iBS, 1991–2020

retail trade, wholesale trade, the construction sector and information service activities. Real estate activities constitute the least affected industry (− 0.1 percent).

## 2.3 Simulating distributional effects

In the next step of our analysis, we translate our estimated labor demand responses into distributional effects using a microsimulation model and household level data.

**The IAB microsimulation model** Specifically, we make use of the IAB microsimulation model (IAB-MSM). The IAB-MSM contains a detailed implementation of the German tax and benefit system. It is based on data from the Socio-Economic Panel (SOEP), a representative yearly household panel study (Goebel et al. 2018). The IAB-MSM simulates the disposable income for each household under varying tax and transfer rules. A detailed description of the calculation of a household's needs and income in the IAB-MSM is provided by Bruckmeier and Wiemers (2011).

We analyze the first-round distributional and fiscal effects of the COVID-19 pandemic on income changes at the household level. The effects are measured as the difference in simulated equivalized gross and net household incomes before and after the COVID-19 labor demand effects, where we denote the pre-crisis scenario as *Baseline*. For the baseline simulation we use the legal status as of January 2020. Deductions from gross wage income and means-tested benefits are simulated. Other income variables, e.g., capital income and pensions, are taken from the survey information. For the tax-benefit simulation, the statutory regulations are implemented as far as possible, whereby information on socio-demographic and regional variables, the income of individuals and households, and current and past worked hours provided in the SOEP are used. Because the tax-transfer module of the IAB-MSM also employs retrospective information to compute the net household income, we require two consecutive waves of the SOEP. For this paper, we employ the SOEP waves 2016 (baseline period $t$) and 2017 ($t+1$, used to obtain retrospective information for period $t$). The data are uprated to the year 2020 through the adjustment of wage and price indicators with the respective growth rates for the period 2016 to 2020.

After sample selection, approximately 15,800 individuals aged 17 and older in 9,300 households remain for use with the IAB-MSM. The most important reasons for excluding households from the simulation sample are missing interviews of persons living in the household (approximately 3,400 households), refugee households (approximately 2,300 households), and missing interviews in wave $t+1$, which represent approximately 1,800 additionally excluded households.[13] To account for the exclusion of households, we adjust the population weights supplied with the SOEP data using reweighting techniques such that the final sample used for the distributional analysis ("distributional sample") is representative for the full population.

Table A1 in the Appendix assesses the quality of the IAB-MSM by comparing the most important simulated fiscal quantities (income taxes, social security contributions, social benefits) to administrative data. Differences may arise from lacking representativeness of the database, non-observable household characteristics, and from errors in the tax-benefit

[13] As the IAB-MSM is not a dynamic model, the inclusion of refugees recently immigrated to Germany would lead to an underestimation of the labor market participation of this group. Brücker et al. (2018) estimate that 28 percent of the refugees who moved to Germany from war and conflict areas after 2014 have taken up employment subject to social insurance contributions by June 2018.

calculation. Table A1 shows that the IAB-MSM is able to replicate the totals from the administrative data with sufficient accuracy.

We perform the distributional analysis by linking the estimated labor demand effects from Section 2.2 with SOEP data at the household level. To achieve this, we assign individuals in the SOEP – both employees and self-employed persons – to the 648 input-by-industry cells used in the prediction of the labor demand effects. Approximately 5,000 persons with income from either dependent employment or self-employment can be assigned to the cells in this way. We calibrate the survey weights of these individuals so that they correspond to 28.8 million individuals, which is the number of people working in the 2-digit NACE industries covered by the iBS according to statistics of the Federal Employment Agency in April 2020. This figure includes approximately 22.9 million regular workers, 3.1 million marginally employed and 2.8 million self-employed persons. The households to which these 5,000 persons belong form a subsample of the 9,300 households used for the distributional analysis. This means that only the 5,000 persons in the former sample are (potentially) affected by the pandemic-induced labor demand shock ("shock sample"). However, our distributional analysis also accounts for several non-employment discretionary policy measures that the German government adopted in the course of the COVID-19 pandemic. These measures potentially affect the incomes of not only households in our shock sample, but all 9,300 households in our distributional sample. We assume that for all persons in the shock sample, the expected relative decline in working hours corresponds to the estimated average labor demand effect in that cell. We also make the assumption that the labor demand shock will have no effect on gross hourly wages, which implies that gross monthly wages will fall in proportion to the reduction in working hours. We simulate distributional effects on both gross and net incomes to capture the dampening effect of the policy responses to the crisis and other features of the tax-benefit system.

**Individual working hour losses** To simulate the effects of the COVID-19 pandemic on the income distribution, we translate the cell-specific average relative working time reductions predicted by our labor demand model (Section 2.2) into person-specific realizations of working hour losses. We carry out this translation by having all potentially affected persons – all regular workers, marginally employed, and self-employed persons that can be linked to one of the 648 labor demand cells – repeatedly play a three-stage "COVID-19 lottery" (see Fig. B1 in the Appendix).

In the first stage of this lottery, individuals draw from a uniform distribution[14] to establish whether they experience a loss in working hours or whether they continue working at their pre-crisis observed working hours (outcome "Unaffected", see Fig. B1). Based on data from the Federal Employment Agency, we calibrate the probability of being unaffected to 67.7 percent. This probability reflects the proportion of people who neither suffered a reduction in working hours nor became unemployed out of all people working in one of the 648 labor

[14] Note that using a uniform distribution in the first and the second stage of the lottery is a simplifying assumption, because the probabilities of not experiencing a loss in working hours (first stage of the lottery) as well as becoming unemployed (second stage of the lottery) likely depend on individuals' personal characteristics. The primary reason for introducing the first two stages of the lottery is to ensure that the expected share of individuals not affected by the crisis and the inflow of individuals into unemployment matches the aggregate data of the Federal Employment Agency. However, in the third stage of the lottery, which determines the size of the relative working hours loss, individual characteristics are taken into account because the expected relative loss of working hours varies with the industry, the contract type, the skill level and the age group of workers.

demand cells covered by the iBS in April 2020, i.e., at the current peak of the pandemic in Germany.

If individuals reach the second stage of the lottery, they draw again from a uniform distribution to establish whether they become unemployed (outcome “Unemployed”) or whether they work a reduced number of hours with short-time work compensation (outcome “Hours Reduction”), where we take into account that marginally employed and self-employed are not entitled to short-time work compensation. For each input-by-industry cell, we calibrate the probability of becoming unemployed such that two conditions are fulfilled: On the one hand, the probabilities of becoming unemployed are proportional to the expected average loss of working hours in a given input-by-industry cell. On the other hand, they are calibrated in such a way that the aggregated increase in expected unemployment amounts to approximately 144,000 individuals. The latter is an estimate of the Federal Employment Agency (2020) for the effect of the COVID-19 pandemic on the average flow from employment into registered unemployment between April 2020 and September 2020 (Federal Employment Agency 2020). The average conditional probability across all input-by-industry cells to become unemployed is 1.56 percent.[15]

If individuals reach the third stage of the lottery, they randomly draw realizations for a) the relative monthly average decrease in working hours and b) the number of months they will be affected by this decrease in 2020. To implement these realizations for the post-crisis situation, we consider two extreme scenarios and an intermediate scenario, which differ in their assumptions for translating the labor demand effect into hours losses (and therefore gross wage losses) at the household level.

The extreme post-crisis scenarios follow the approach of Bargain et al. (2012). In the extensive scenario (*Extensive*), individuals stochastically suffer a reduction in working hours of either 0 percent or 100 percent.[16] In contrast, the intensive scenario (*Intensive*) implies a deterministic adjustment of working hours. Here, all individuals from the same input-by-industry group suffer the same relative hours reduction. Crucially, in both extreme scenarios, we ensure that the expected relative loss in working hours (calculated over all three stages of the game) for each individual belonging to a specific input-by-industry cell corresponds to the cell-specific labor demand effect from Section 2.2. Additionally, both extreme scenarios assume that the decrease in working time (if it occurs) will last for 10 months, which is the maximum possible duration of a loss of working time in 2020 related to COVID-19, given that the effects of the crisis are felt since March 2020.

While these polar cases are useful for estimating the range of effects on the income distribution, neither of them is realistic. Statistics of the Federal Employment Agency from April 2020 show that the loss of working hours caused by the COVID-19 pandemic varies greatly among those affected by short-time work (see Table 4). Thus, there is no empirical justification for the assumption that the reduction in working time applies only at the extensive or the intensive margin.

Due to the non-linearity of the German tax-benefit system, both extreme scenarios might deliver biased results for the distributional analysis. For example, as the German income tax schedule is progressive, the more the reduction in working hours is concentrated on employees, the smaller the decline in income tax revenues will be. As a consequence, it is

[15]The probability is conditional on reaching the second stage. The unconditional probability is $1.56\% \times 32.3\% = 0.5\%$. Accordingly, an affected individual reaches the third stage of the game with the unconditional probability $(100\% - 1.56\%) \times 32.3\% = 31.8\%$

[16]Note that the *Extensive* scenario is a special case in that it allows for the possibility of not being affected by a loss in working hours, despite having reached the third stage of our lottery.

**Table 4** Working hours reduction for employees on short-time work

| Relative change in hours (in percent) | ≤ 25 | 26–50 | 51–75 | 76–100 | 100 |
|---|---|---|---|---|---|
| Share of short-time workers (in percent) | 21.3 | 31.3 | 15.4 | 8.0 | 24.1 |

The table shows results from a survey conducted by the Institute of Employment Research (IAB) in May 2020. Source: Kruppe and Osiander (2020)

important to adequately capture individual heterogeneity both in terms of working hours reductions and months of being affected. While we have estimates of the average relative reductions in working hours in each of our 648 input-by-industry cells in 2020, we do not observe for any of our cells the joint distribution of relative working time losses and of the number of months in which people are affected by working time losses. To tackle this problem and to arrive at a more realistic estimate of the distributional consequences, we propose a more appropriate approach described next.

**Maximum entropy method** In our third scenario (*MaxEntropy*), we use the principle of maximum entropy (Jaynes 1957a, b) to derive bivariate discrete distributions of relative working hours reductions and the months of being affected by the labor demand shock for each input-by-industry cell. The entropy of a random variable is defined as the average level of information in the variable's possible outcomes (Shannon 1948).[17] The principle of maximum entropy states that given precisely specified prior data, the probability distribution that best reflects our limited knowledge is the one with largest entropy.

More specifically, for each individual[18] who reaches the third stage of the game, we take $R$[19] draws $(\widetilde{H}, M)_r \in \mathcal{S}, r = 1, \ldots, R$ of relative working hour reductions, $\widetilde{H} \in \mathcal{H}$, and months of being affected by the shock, $M \in \mathcal{M}$, from a discrete bivariate probability distribution $f(\widetilde{H}, M)$ with support $\mathcal{S} = \mathcal{H} \times \mathcal{M}$ where $\mathcal{H} = \{0.1,\ 0.2, \ldots, 1\}$[20] and $\mathcal{M} = \{1,\ 2, \ldots,\ 10\}$.[21] Following the principle of maximum entropy, we determine the probability distribution $\mathbf{p} := \{p_s | s \in \mathcal{S}\}$ of $f(\widetilde{H}, M)$ by maximizing the entropy function $\mathscr{H}(\mathbf{p}) = -\sum_{s\in\mathcal{S}} p_s \ln(p_s)$ subject to the constraints $\sum_{s\in\mathcal{S}} p_s = 1$, i.e., the probabilities

[17] The basic insight of information theory is that different probability distributions of a random variable contain different amounts of "uncertainty" or, equivalently, "information". For example, if we observe the outcome of tossing a fair coin, our uncertainty about that outcome is reduced "very much", i.e., the outcome contains "a lot" of information. If, on the contrary, we know that the result of a coin toss is always "heads", then the probability distribution of this random variable contains no uncertainty, and observing an outcome of tossing that coin contains no information. To operationalize this idea, a measure for "uncertainty" or "information" is needed. Of all conceivable ways of measuring uncertainty, entropy is the only measure that satisfies three intuitive properties that such a measure should have: i) The measure should be continuous, i.e., a small change in a probability should not result in a massive change of uncertainty; ii) the measure of uncertainty should increase as the number of possible events increases; and iii) the measure should be additive, i.e., if we measure the uncertainty about two possible events A and B and then the uncertainty about two other events C and D, the sum over the four combinations A/C, A/D, B/C, B/D should be the sum of the separate uncertainties. For a non-formal, intuitive introduction to information theory and its ties to Bayesian statistics, see, e.g., McElreath (2020).

[18] To simplify the notation, we omit indices for individuals and their respective input-by-industry cell.

[19] We set the number of draws for both the MaxEntropy and the Extensive scenario to $R = 100$. Increasing the number of draws does not substantially alter the results.

[20] We considered smaller step sizes for the relative working hours reductions, e.g., 5 percent instead of 10 percent steps, but this had no substantial impact on the results.

[21] Since the impact of the pandemic only started to occur in March 2020, we consider a maximum period of 10 months to be affected by the labor demand shock in 2020.

for each input-by-industry distribution need to sum to one, and $\sum_{s \in \mathcal{S}} \frac{p_s \cdot \widetilde{H} \cdot M}{12} = \mu$, i.e., the expected value of the distribution has to equal the predicted mean average reduction in working hours $\mu$ of the specific input-by-industry cell in 2020. We proceed by numerically solving the first-order conditions of the corresponding Lagrangian

$$\mathcal{L}(\mathbf{p}, \lambda_0, \lambda_1) = -\sum_{s \in \mathcal{S}} p_s \ln(p_s) - \lambda_0 \left( \sum_{s \in \mathcal{S}} p_s - 1 \right) - \lambda_1 \left( \sum_{s \in \mathcal{S}} p_s \frac{\widetilde{H} \cdot M}{12} - \mu \right) \tag{6}$$

for the probabilities $p_s$ and the Lagrangian multipliers $\lambda_0$ and $\lambda_1$.

We implement the MaxEntropy scenario, which – to the best of our knowledge – has not been applied in this context before, as our baseline for the translation of predicted labor demand responses from input-by-industry cells to individual declines in working hours. However, in Section 3, we report the distributional and fiscal effects of the COVID-19 pandemic for all three scenarios in order to show the range of effects to be expected under extreme assumptions regarding the reduction of working time.

**Non-take-up** In all three scenarios, non-take-up of means-tested benefits is accounted for along the lines of Pudney et al. (2006) and Wiemers (2015). For this purpose, we estimate a binary probit model with random effects, where the dependent variable indicates whether the household has claimed any means-tested benefit, i.e., basic income, housing benefits, or child benefit supplement. The estimation sample is conditional on the simulated eligibility for at least one means-tested benefit, where the eligibility simulation is based on the IAB-MSM. The estimation uses the SOEP waves 2014 to 2016. We employ a stochastic simulation approach (Pudney et al. 2006) for the out-of-sample prediction of take-up in 2020, where the predictions of take-up are conditional on the observed take-up behavior in 2016.

### 2.4 Modeling policy responses to the crisis

**Short-time work** Short-time work has arguably been the most important policy instrument to prevent layoffs in the current COVID-19 pandemic. The government supports the companies financially a) by paying employees short-time work compensation (SWC) for the hours they have not worked and b) by reimbursing the company for social security contributions (except for unemployment insurance) provided that certain minimum requirements are met: First, only companies in which at least one employee subject to social insurance contributions is employed are eligible for SWC. Second, since March 2020, the loss of working hours must affect at least 10 percent (previously at least 33 percent) of the employees with a loss of earnings of more than 10 percent each. Third, since SWC is a benefit paid by the social insurance system, only employees who are subject to social insurance contributions can receive it. Since March 2020, temporary workers who were previously excluded from receiving SWC have also been entitled to SWC. In contrast to the financial crisis in 2008/09, many other sectors besides manufacturing are massively affected by the economic consequences of the pandemic. As a consequence, the German government has simplified access to short-time work compensation - limited until 31 December 2021. SWC can be drawn for up to 12 months, and under certain circumstances for up to 24 months.

The SOEP contains only limited information about the companies in which individuals work. In particular, we do not know whether the companies meet the minimum requirements to be entitled to SWC. In our simulations, we assume that these minimum requirements are always met. Especially during the months March to May 2020, i.e., at the peak of the pandemic, this assumption is highly plausible.

The latest preliminary statistics from the statistics of the Federal Employment Agency forecast 2.6 million short-time workers in August 2020, a significant decrease compared to the 6.0 million short-time workers in April 2020, the peak of the COVID 19 crisis so far. The pre-crisis number of short-time workers in February 2020 was only 134,000.

The amount of SWC is similar to the unemployment benefit, i.e., 60 percent of the last net wage (67 percent if children are present in the household). In addition, the amount of SWC has been staggered over time. SWC can increase to 70 (77) percent after three months and 80 (87) percent after six months of receiving SWC. In our modelling of SWC, we take these increases into account. Moreover, we also consider that a proportion of people whose working hours fall to zero due to the pandemic do not receive SWC, but unemployment benefits.

Since employees in Germany have to accept considerable losses of income during short-time work, the trade unions have long been trying to increase the governmental SWC by means of collectively agreed supplementary SWC (SSWC). Therefore, there are currently a large number of collective bargaining agreements in place that reduce or even completely compensate employees' loss of income in the event of short-time work through SSWC. These agreements differ with regard to, e.g., the amount of the collectively agreed SSWC, the question of whether the supplement is based on net or gross pay, and the question of who finances the supplement (Schulten and Müller 2020). Additionally, a recent survey from Hans-Böckler-Stiftung (2020) shows that people who work in a company with a collective bargaining agreement are significantly more likely (54 percent) to receive SSWC than people who are not paid under a collective agreement (31 percent). Therefore, in our simulations, we randomly assign SSWC to individuals affected by the COVID-19 shock according to these probabilities. With the combination of SWC and SSWC, short-time workers typically reach between 80 and 100 percent of their regular net income. Since we have no information on the individual net replacement rate of SSWC, we assume that it will increase the net income of all SSWC recipients to 90 percent of their original net wages.

**Discretionary non-employment policy measures** In addition to strengthening the SWC, the government has adopted a number of discretionary non-employment measures to relieve households at least partially from the financial impact of the COVID-19 pandemic. These measures, which we also consider in our simulations, are:

- Child bonus: Families receive a one-off child bonus of 300 Euros for each child that is entitled to the regular not means-tested child benefit in at least one month in 2020. While the bonus is not credited against means-tested benefits such as basic income support, it is offset against the income tax benefit resulting from the child allowance.
- Support for single parents: In order to provide targeted support for single parents, the special income tax allowance for single parents is increased from 1,908 Euros to 4,008 Euros for the years 2020 and 2021.
- Emergency child benefit supplement ("Notfall-KiZ"): For families with children who lose earnings due to the COVID-19 pandemic, an ''emergency child benefit supplement'' came into force on 1 April 2020. In addition to some changes to make it easier to apply for the child benefit supplement, parents no longer have to provide information on assets if they do not have substantial assets. We interpret "substantial assets" in the sense of the housing benefit regulations, i.e., considerable assets do not exist if they do not exceed a value of 80,000 Euros for the head of household plus 30,000 Euros for each additional person in the household. This regulation is valid until 30 September 2020.

- Simplified access to basic income support: Anyone who submits an application for basic income support benefits between 1 March and 30 September 2020 and declares that they do not have substantial assets may keep any savings. In addition, expenditure on housing and heating during the first six months of receiving benefits is recognized at the actual amount. Normally, costs for housing and heating are only reimbursed up to a "reasonable" level, which is determined by the size of the household and local costs of housing.

We cannot consider several minor measures in our simulations due to a lack of information in our data. Those measures include a continued payment of wages due to school and kindergarten closures and an adjustment of the parental allowance. However, compared to the measures considered in the simulations, especially the SWC, fiscal expenditures for these minor measures are so low that they are unlikely to affect the results of our distribution analyses.

## 3 Effects on the income distribution

For the distribution analysis, we compute relative changes in equivalized household incomes between the pre-crisis baseline scenario and the alternative post-crisis scenarios focusing on different income distributions: gross labor income, disposable income of the working population, disposable income of the total population, and disposable income of the total population accounting for discretionary non-employment responses to the pandemic. The simulated income changes relate to the entire year 2020, whereby the predicted labor demand changes are based on employers' output expectations for 2020 measured in April, June or September 2020. The use of different reference dates for the demand projection gives an impression of the dynamics of the crisis.[22] In the following, we interpret the results for our preferred scenario (*MaxEntropy*). At the end of this section, we briefly discuss the differences in the results between the three scenarios.

**Gross labor income** First, we examine the effect on the gross labor income of households with at least one employee or self-employed working in sectors negatively affected by the crisis. Figure 2 presents the relative changes by (pre-crisis) gross labor income decile groups. Based on the output information as of September 2020, the most recent month for which survey data is available, we find an average decline in gross income by − 3 percent. All households across the entire distribution suffer from income losses. We see the largest relative negative effects in the first (−4.3 percent) and eighth decile group (−3.6 percent) and the lowest income losses in the fifth (− 2.4 percent) and third decile (− 2.6 percent). Overall, gross wage inequality slightly increases.[23] The impact on all income groups is also consistent with the projected employment effects, which affect well-paid jobs in the manufacturing sector as well as low-paid jobs in the service sector.

Figure 2 reflects that the companies' output prospects for 2020 improved in the months after April 2020. Based on output growth expectations measured in April 2020, the peak of

[22] In addition, we present the simulated fiscal effect on the government budget. To this end, Table A2 in the Appendix shows the difference in simulated expenditure on social benefits and the income tax and social security contributions in 2020 between the pre-crisis and post-crisis scenarios.

[23] The Gini coefficient with respect to gross labor income increases by approximately 0.3 percentage points based on September data. For example, Palomino et al. (2020) also find an increase in gross wage inequality in Germany.

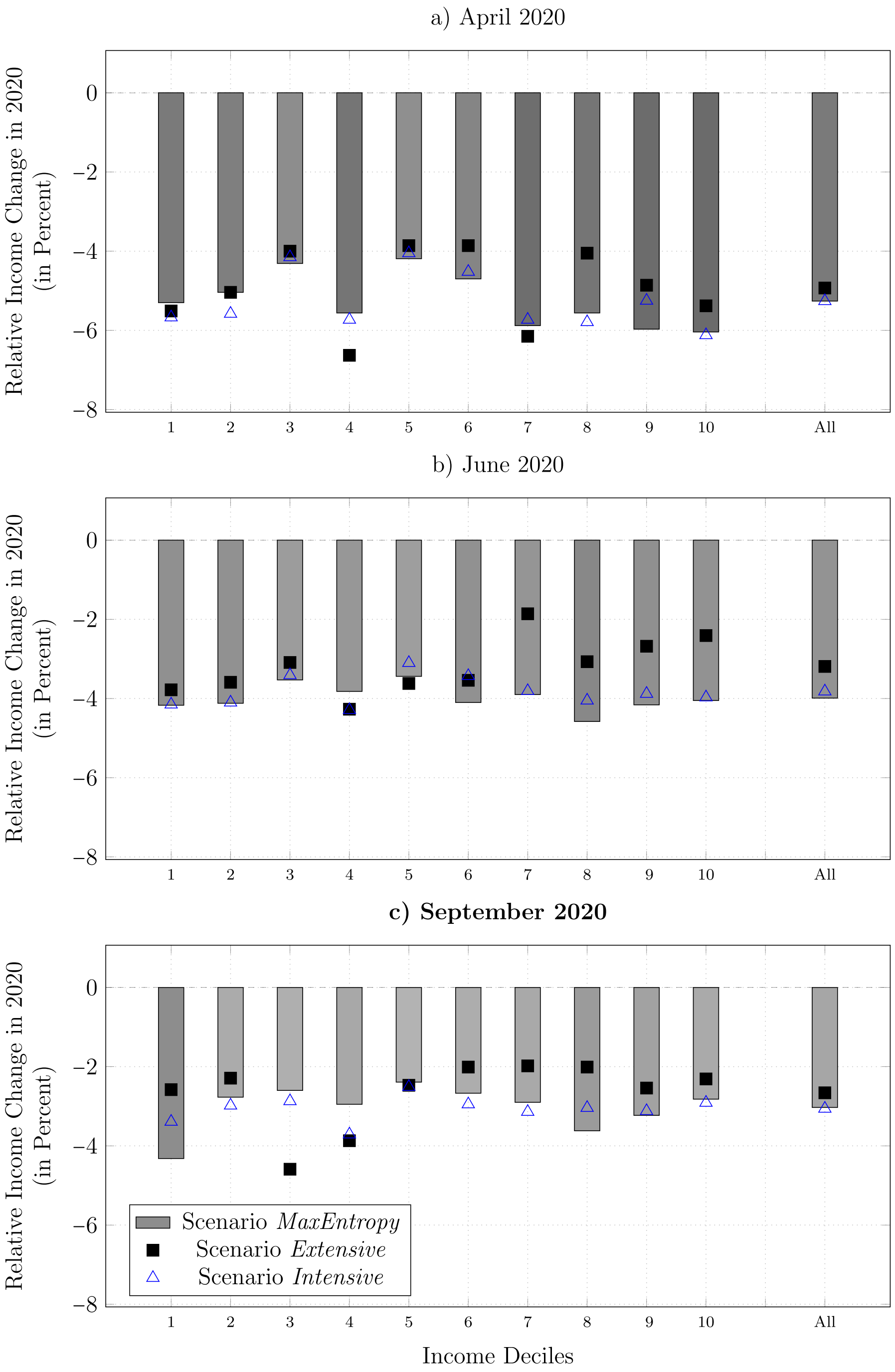

**Fig. 2** COVID-19 effects on gross income by income deciles, affected population. Relative change in mean household equivalent gross income by gross income decile compared to the baseline for the subpopulation of households that are potentially affected by the crisis. The equivalent income is calculated based on the modified OECD scale. All = Mean change over deciles. Source: IAB-MSM

the crisis so far, we simulate an average decline in gross income by − 5.3 percent. Based on information gathered in June we simulate an average income loss of − 4 percent. This reflects the dynamic development of the crisis and the associated policy measures. Bauer and Weber (2020) estimate that about 60 percent of the increased inflows into unemployment in April 2020 in Germany could be explained by the containment measures to stop the spread of the virus. The results are therefore extremely dependent on the short-term development of the pandemic, and forecasts based on the first months of the pandemic could deviate significantly from the actual development.

**Net income of the working population** Figure 3 shows the relative net income change for the households with individuals working in the affected sectors. Net income includes not only net labor income but also social benefits with the exception of the discretionary non-employment measures taken by the federal government in reaction to the crisis. Thus, the comparison between Figs. 2 and 3 shows the stabilizing effect of the tax and benefit system (that was in place at the beginning of 2020) on household incomes.

Overall, the loss of income is reduced significantly by 1.9 percentage points to an average reduction in net income to only −1.1 percent (September).[24] Moreover, the tax and transfer system is effective in changing the structure of income losses across the income distribution. Lowest net income losses are experienced in the middle parts of the distribution, the highest income losses are in the first, eighth, ninth and tenth decile groups (− 1.5 percent). The buffering effect of the tax-benefit-system is mainly driven by the SWC (which was already in place before the crisis).

Since SWC is not means-tested, all employees generally benefit regardless of their income level. However, the amount of SWC is limited to the income threshold up to which social security contributions are assessed. As a result, high income groups benefit less from SWC than groups whose income is below this threshold. At the same time, there are groups that are excluded from SWC, mainly self-employed individuals or employees with monthly earnings up to 450 Euro (so called "minijobbers"). These two characteristics of the SWC together imply that the middle income groups benefit most from SWC in relative terms, which is indicated by Fig. 3.

**Net income of all households** To examine the effect of the employment shocks on the overall income distribution, we extend the analysis to the entire population. Figure 4 presents the net income changes for the decile groups based on the equivalent household net incomes of all households, including those exclusively consisting of non-working individuals. In relation to the income of the total population, the negative income effect is further reduced to − 0.6 (September forecast), − 0.7 (June forecast) and − 0.9 (April forecast) percent, respectively. Because a disproportionately large number of inactive people, e.g., recipients of means-tested benefits, are represented in the lower deciles, the average relative income losses in the lower decile groups fall significantly compared to Fig. 2. Based on the September forecast, we simulate very small effects on net income of the first and second decile (− 0.24 percent each).

**Discretionary non-employment policy measures** In the last step, we include the discretionary non-employment policy measures described in Section 2.3 in the calculation of disposable incomes (see Fig. 5). Once these measures are taken into account, the overall

[24] Based on the April and June forecasts, we simulate an average decline in net income of − 1.6 and − 1.3 percent, respectively.

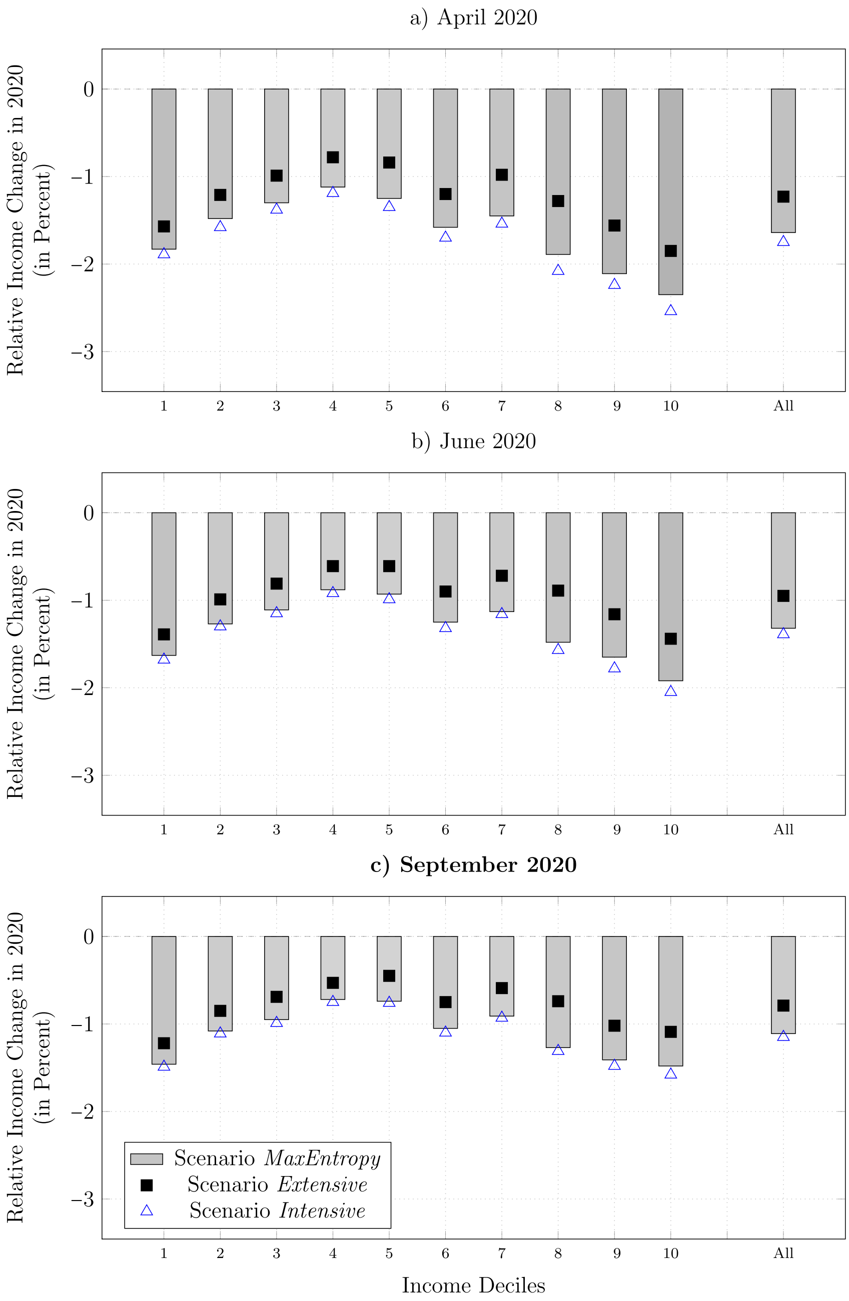

**Fig. 3** COVID-19 effects on net income by income deciles excluding discretionary non-employment policies, affected population. Relative change in mean household equivalent net income by net income decile compared to the baseline for the subpopulation of households that are potentially affected by the crisis. The equivalent income is calculated based on the modified OECD scale. Discretionary non-employment policy measures (child bonus, support for single parents, emergency child benefit supplement, simplified access to basic income support) are excluded in the simulation. All = Mean change over deciles. Source: IAB-MSM

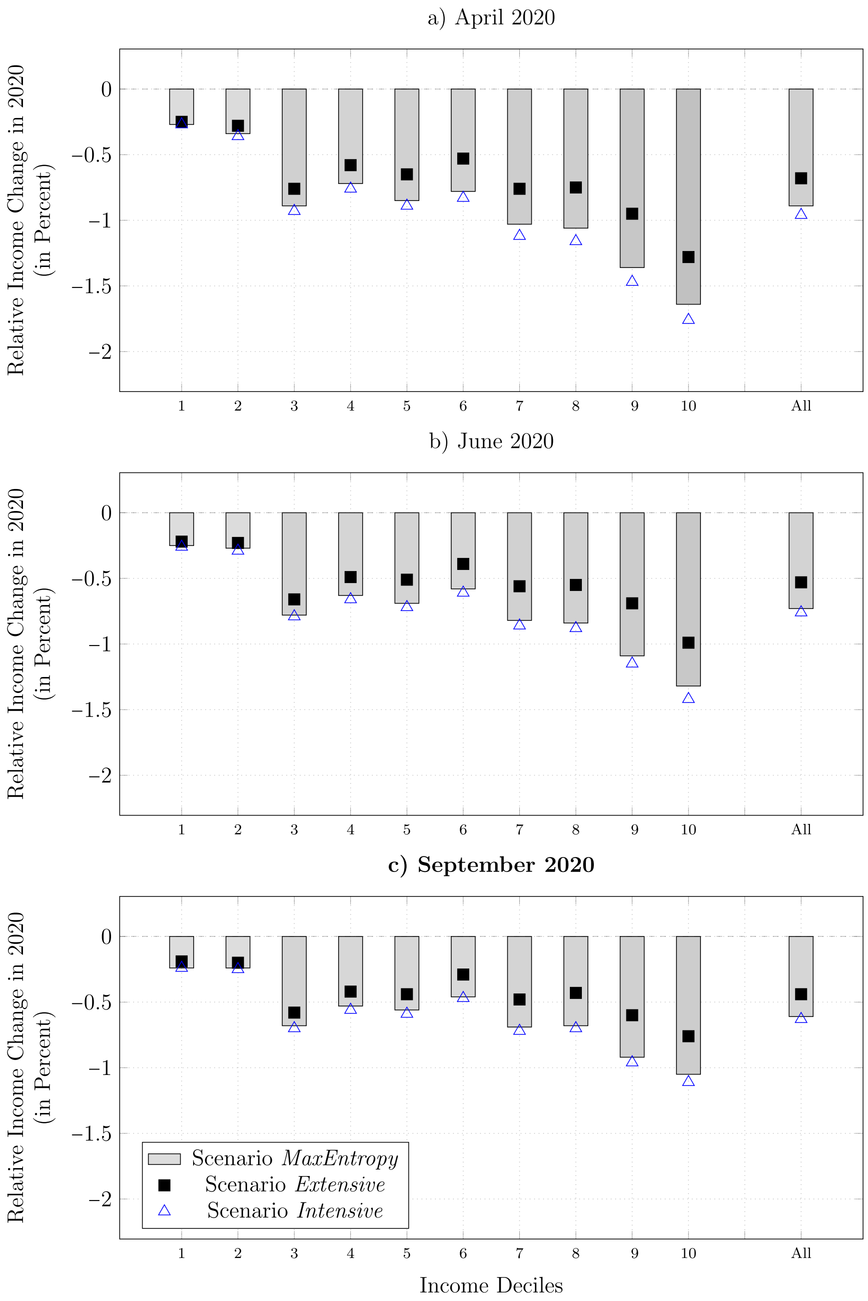

**Fig. 4** COVID-19 effects on net income by income deciles excluding discretionary non-employment policies, overall population. Relative change in mean household equivalent net income by net income decile compared to the baseline for the overall income distribution. The equivalent income is calculated based on the modified OECD scale. Discretionary non-employment policy measures (child bonus, support for single parents, emergency child benefit supplement, simplified access to basic income support) are excluded in the simulation. All = Mean change over deciles. Source: IAB-MSM

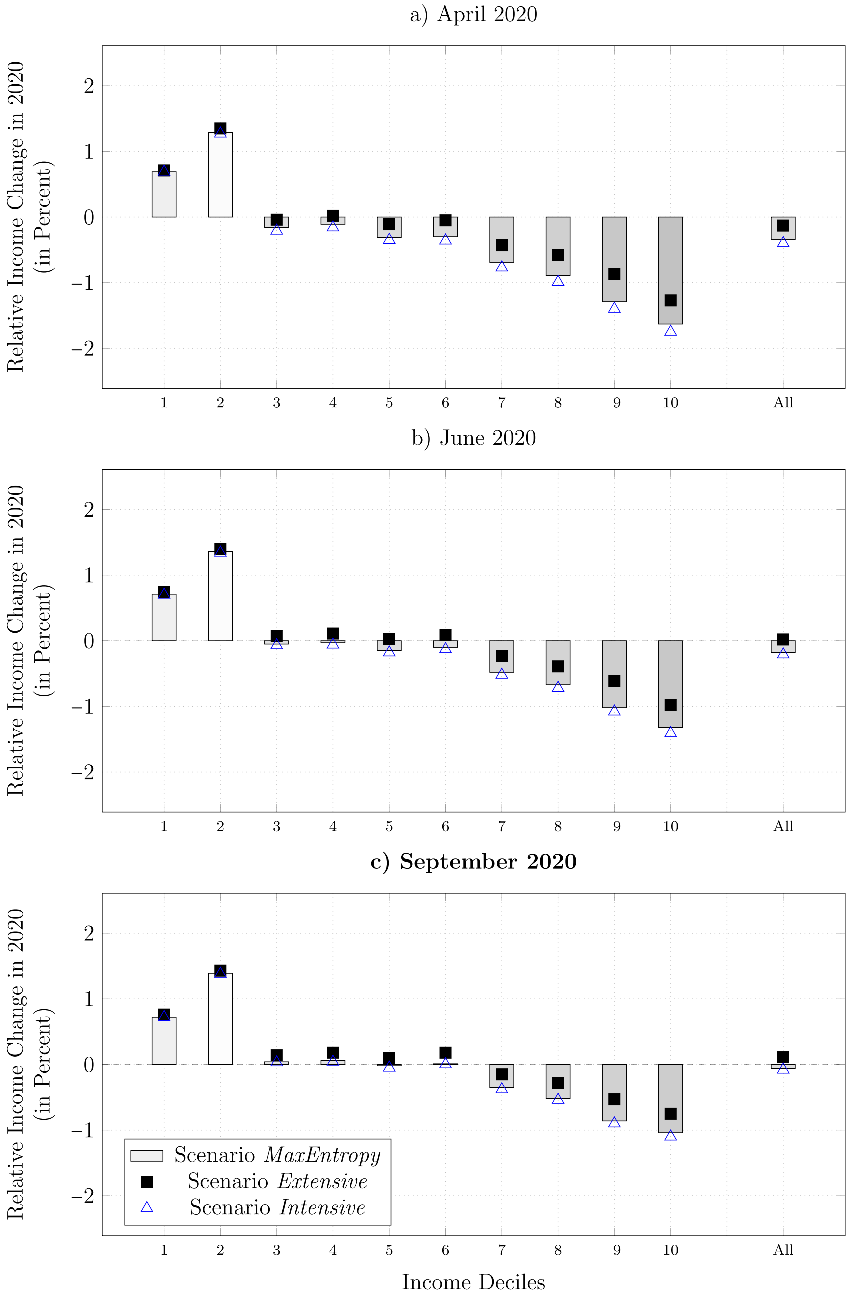

**Fig. 5** COVID-19 effects on net income by income deciles including discretionary non-employment policies, overall population. Relative change in mean household equivalent net income by net income decile compared to the baseline for the overall income distribution. The equivalent income is calculated based on the modified OECD scale. Discretionary non-employment policy measures (child bonus, support for single parents, emergency child benefit supplement, simplified access to basic income support) are included in the simulation. All = Mean change over deciles. Source: IAB-MSM

effect on household disposable income is reduced to only − 0.06 percent (September forecast), − 0.18 (June forecast) and − 0.34 percent (April forecast). The income effect even turns positive for all forecasts for the first and second decile groups in the June and September forecast, while the effect is almost zero for the middle income groups and remains negative in the decile groups above.

Figure 5 indicates that the negative effect increases with income and the total effect on the income distribution turns progressive. This strong redistributive effect of the discretionary non-employment measures is mainly driven by the child bonus. In quantitative terms, the child bonus is the most important of the non-employment policy measures described in Section 2.3. We simulate additional spending of 5.1 billion euros for the child bonus in 2020. In comparison, the additional expenditure on short-term work due to the crisis (excluding social security contributions) amount to 10.3 billion euros based on the September forecast (see Table A2 in the Appendix). The child bonus is targeted at low and middle income households. Firstly, because unlike the regular child bonus, it is not considered in the means test for social benefits. Secondly, high income earners do not benefit from it because the bonus is offset against the income tax benefit resulting from the child allowance.

The distribution of the income changes across the decile groups is consistent with our findings for the impact on income inequality, although the effect is almost negligible. Based on a constant (pre-crisis) poverty line and the September forecast, we simulate a decrease in the poverty rate by − 0.45 percent (− 0.09 percentage points) and in the Gini coefficient by − 0.95 percent (− 0.29 percentage points) (see Table A3 in the Appendix).

Although the evidence on the distributional effects of the COVID-19 crisis is less developed so far, our results are consistent with the few other studies that suggest similar basic mechanisms behind the distributional effects. First, the overall negative effects on income distribution decrease significantly during the crisis. For example, Brewer and Tasseva (2020) simulate a strong negative initial effect on the net income of all UK households for April 2020 of (− 8 percentage points). In contrast, based on conservative assumptions about the recovery phase, Bronka et al. (2020) find a decline of only 1 percentage point for 2020. Second, short-time work schemes or wage subsidies for employees are the main insurance mechanism in many countries (Konle-Seidl 2020). Germany relies on a well-functioning system of short-time work implemented in the unemployment insurance. At the same time, the benefits that workers can receive are relatively high in an international comparison and therefore, and contrary to other countries, a large part of the income shock is offset through the welfare system (Almeida et al. 2021; Figari and Fiori 2020). As the SWC schemes are usually not means-tested, but paid up to a maximum ceiling, households from the middle income groups benefit particularly from these benefits. Third, while the employment related income shock affects all households along the (labor) income distribution, the total income effect seems to be progressive, with income gains in the lower tail of the income distribution and a reduction in the Gini-coefficient and the poverty rate. This is mainly driven by non-employment benefits, e.g., the child bonus in Germany or the increased generosity of the Universal Credit in the UK (Bronka et al. 2020), or by reduced housing and work related expenses (O'Donoghue et al. 2020).

Our simulation results show the following findings with regard to the three implemented post-crisis scenarios. For most of the results, scenario *Extensive* is the upper bound and scenario *Intensive* the lower bound for the simulated negative income effect on the decile groups. However, differences are small in most cases. Hence, it does not seem to be important whether we distribute the full income shock to a subgroup in a sector or distribute a small income shock to all employees in that sector, as is done in the two extreme scenarios. The differently implemented income shocks are averaged out within deciles to some extent and we observe only minor differences between the three scenarios. This holds especially for the lower income groups. For example, for the first two deciles we find an overlap between the three scenarios. This is also explained by a higher share of non-affected (non-employed) people living in these low-income households. Furthermore, moving from Figs. 2 and 3, the difference for these two extreme scenarios also decreases for lower income groups and increase for higher income groups due to the increasing non-linearity of the tax and transfer system for higher incomes. For a high monthly gross wage, for example, the (progressive) income tax decreases significantly more for a 100 percent income shock than for a low loss of income. Accordingly, the differences between the scenarios are more pronounced for the fiscal effects, for example, if one considers the effects on the income tax or expenditure on short-time work benefits, which both depend on the individual level of income loss.

## 4 Conclusion

Governments around the world use drastic interventions to slow down and control the COVID-19 pandemic. Both the pandemic itself as well as the containment measures affect economic activities in almost all sectors and have a dramatic economic impact. The high dynamics of the crisis and the high level of uncertainty in the economy pose an unprecedented challenge for policy makers to take the appropriate countermeasures to reduce the economic damage. The assessment of the consequences of the crisis and the effects of the countermeasures based on the latest economic developments is therefore central to overcoming the crisis.

In this paper, we propose a novel method to quantify the short-term effects of a macroeconomic shock on labor demand and on the income distribution in real-time. We provide an application to Germany in the context of the COVID-19 pandemic. As such, this study is also the first to estimate the distributional effects of the COVID-19 crisis for Germany. Our approach combines different data sources and economic models in a novel way and can be updated regularly: an output forecast building on VAR models estimated on monthly firm-level survey data, a structural labor demand model based on cost minimization and a microsimulation model. In order to show the dynamics of the crisis and its impact on the results obtained, we report results that are based on information collected at three different times: April, June and September 2020. As a novelty, the estimated macro and labor demand effects are linked to household micro data based on the principle of maximum entropy. This principle, which has not yet been applied in previous studies in this context, offers the necessary flexibility to take into account sector-specific distributions of relative working hours changes as well as the months of being affected by the crisis. This feature is particularly important during the first phase of the crisis when employment responses takes place predominantly via the intensive rather than the extensive margin. The tools and methods developed in this paper are also applicable to many other countries in which labor market and distributional microdata is not available in real-time.

Our findings show a significant decline in output in almost all industries in 2020. This output shock translates into a noticeable reduction in both, labor demand and gross labor income. All working households across the income distribution suffer from income losses. The tax benefit system and especially the short-time work program act as an important automatic stabilizer as expected losses in disposable income are significantly reduced. As a disproportionately large number of inactive people, e.g., recipients of means-tested benefits, are represented in lower deciles, the average relative income losses in the lower decile groups become smaller when including non-employed households in the analysis. Finally, when accounting for the discretionary non-employment policy measures enacted as a response to the recession, the average effect on the income distribution turns progressive as the first two decile groups actually gain income, while the middle income groups are almost not affected and the upper parts still lose income.

A comparison of our results based on information collected in April, June and September 2020 shows that business expectations improved quickly after April 2020 and hence, estimated negative effects on the income distribution declined significantly by September 2020. While our results presented up to September still suggest that the recovery of the German economy is following a V-shape, the strong increase in infections in October 2020 in Germany and Europe already challenges this finding. Hence, the analysis of the economic impacts of the crisis and a continual evaluation of policy responses remains highly topical.

**Supplementary Information** The online version contains supplementary material available at https://doi.org/10.1007/s10888-021-09489-4.

**Funding** Open Access funding enabled and organized by Projekt DEAL.

**Open Access** This article is licensed under a Creative Commons Attribution 4.0 International License, which permits use, sharing, adaptation, distribution and reproduction in any medium or format, as long as you give appropriate credit to the original author(s) and the source, provide a link to the Creative Commons licence, and indicate if changes were made. The images or other third party material in this article are included in the article's Creative Commons licence, unless indicated otherwise in a credit line to the material. If material is not included in the article's Creative Commons licence and your intended use is not permitted by statutory regulation or exceeds the permitted use, you will need to obtain permission directly from the copyright holder. To view a copy of this licence, visit http://creativecommons.org/licenses/by/4.0/.

## Affiliations

**Kerstin Bruckmeier[1] · Andreas Peichl[2] · Martin Popp[1] · Jürgen Wiemers[1] · Timo Wollmershäuser[2]**

Kerstin Bruckmeier
kerstin.bruckmeier@iab.de

Andreas Peichl
peichl@econ.lmu.de

Martin Popp
martin.popp@iab.de

Timo Wollmershäuser
wollmershaeuser@ifo.de

1 Institut für Arbeitsmarkt- und Berufsforschung (IAB) der Bundesagentur für Arbeit (BA), Regensburger Straße 104, 90478 Nürnberg, Germany

2 ifo Zentrum für Makroökonomik und Befragungen, Poschingerstraße 5, 81679 München, Germany

## Electronic supplementary material

Below is the link to the electronic supplementary material.

(PDF 231 KB)
