# Supplementary material for: Distributional effects of macroeconomic shocks in real-time: A novel method applied to the COVID-19 crisis in Germany
Source: J Econ Inequal. 2021 Sep 20;19(3):459–87. doi: 10.1007/s10888-021-09489-4 (PMC8452132; doi:10.1007/s10888-021-09489-4)
Supplement: Supplementary file 1 — (PDF 231 KB) [file 10888_2021_9489_MOESM1_ESM.pdf]

A Appendix: Tables

Table A1: Comparison to Administrative Data, Revenues and Expenses, 2016 (in Million Euro)

	**Simulated**	**Reference**	**Source**
**Revenues**			
Income Taxes[a]	273,823	238,659	Destatis, 2018, p. 279
Pension Insurance Contributions[b]	198,684	195,517	Destatis, 2018, p. 236
Unemployment Insurance Contributions[b]	30,514	32,399	Destatis, 2018, p. 236
Health, Nursing Care Insurance Contributions[b]	203,523	210,819	Destatis, 2018, p. 236
**Expenses**			
Unemloyment Benefit II (Standard Requirements)	14,709	14,902	BA, 2016, Table 1
Unemloyment Benefit II (Accomodation Costs)	15,710	14,078	BA, 2016, Table 1
Housing Benefits	810	1,147	Destatis, 2020
Child Benefit	39,518	35,210	FamKa, 2016, p. 5
Supplementary Child Allowance	417	325	FamKa, 2016, p. 5

NOTE. — Simulated fiscal revenues and expenditures (in million Euro) in 2016 compared to administrative data. [a]Total from wage tax and assessed income tax. [b]Includes both, employer and employee contributions. Source: IAB-MSM, SOEP v34.

Table A2: Fiscal Effect (in Million Euro)

**Scenario**	**MaxEntropy**			**Extensive**			**Intensive**		
Forecast Period	4/2020	6/2020	9/2020	4/2020	6/2020	9/2020	4/2020	6/2020	9/2020
**Increase in Expenditures**									
UB II (Standard Requirements)	176	160	**138**	201	183	**167**	182	168	**142**
UB II (Accommodation Costs)	383	358	**339**	427	392	**375**	387	363	**343**
Housing Benefits	12	12	**11**	21	20	**18**	12	12	**12**
Supplementary Child Allowance	2	4	**5**	6	6	**6**	1	3	**4**
Bonus Child Benefit	5,081	5,080	**5,080**	5,079	5,078	**5,078**	5,081	5,080	**5,080**
Short-Time Work Compensation	18,266	13,121	**10,280**	23,396	17,704	**14,365**	17,069	12,150	**9,883**
SSC on Short-Time Work Compensation	14,611	10,907	**8,626**	15,184	11,432	**9,243**	14,501	10,666	**8,523**
Unemployment Benefit I	1,103	799	**646**	1,103	799	**646**	1,103	799	**646**
**Decrease in Revenues**									
Income Taxes	14,532	11,122	**8,752**	12,726	9,388	**7,388**	15,020	11,365	**8,991**
SSC Employees	2,033	1,446	**1,196**	2,545	1,945	**1,591**	1,921	1,439	**1,226**
SSC Employers	1,877	1,333	**1,084**	2,287	1,739	**1,414**	1,764	1,312	**1,103**
**Total Fiscal Costs**	58,076	44,341	**36,158**	62,975	48,687	**40,292**	57,040	43,357	**35,953**

NOTE. — Differences in fiscal revenues/expenditures (in million Euro) compared to the baseline. Discretionary non-employment policy measures (child bonus, support for single parents, emergency child benefit supplement, simplified access to basic income support) are included in the simulation. SSC = Social Security Contributions. UB II = Unemployment Benefit II. Source: IAB-MSM.

Table A3: Changes in Gini Coefficient and Poverty Rate

**Scenario**	**MaxEntropy**			**Extensive**			**Intensive**		
Forecast Period	4/2020	6/2020	9/2020	4/2020	6/2020	9/2020	4/2020	6/2020	9/2020
Gini Coefficient: Absolute Difference to Baseline (in Percentage Points)	-0.37	-0.33	**-0.29**	-0.32	-0.28	**-0.25**	-0.39	-0.34	**-0.30**
Gini Coefficient: Relative Difference to Baseline (in Percent)	-1.21	-1.07	**-0.95**	-1.03	-0.90	**-0.81**	-1.26	-1.12	**-0.98**
Poverty Rate: Absolute Difference to Baseline (in Percentage Points)	-0.08	-0.07	**-0.09**	-0.07	-0.09	**-0.10**	-0.07	-0.08	**-0.09**
Poverty Rate: Relative Difference to Baseline (in Percent)	-0.37	-0.35	**-0.45**	-0.35	-0.43	**-0.46**	-0.33	-0.40	**-0.42**

NOTE. — Absolute and relative differences in Gini coefficient and poverty rate compared to the baseline for the overall income distribution. The net equivalent income is calculated based on the modified OECD scale. Discretionary non-employment policy measures (child bonus, support for single parents, emergency child benefit supplement, simplified access to basic income support) are included in the simulation. Source: IAB-MSM.

B Appendix: Figures

Figure B1: Three-Stage COVID-19 Lottery

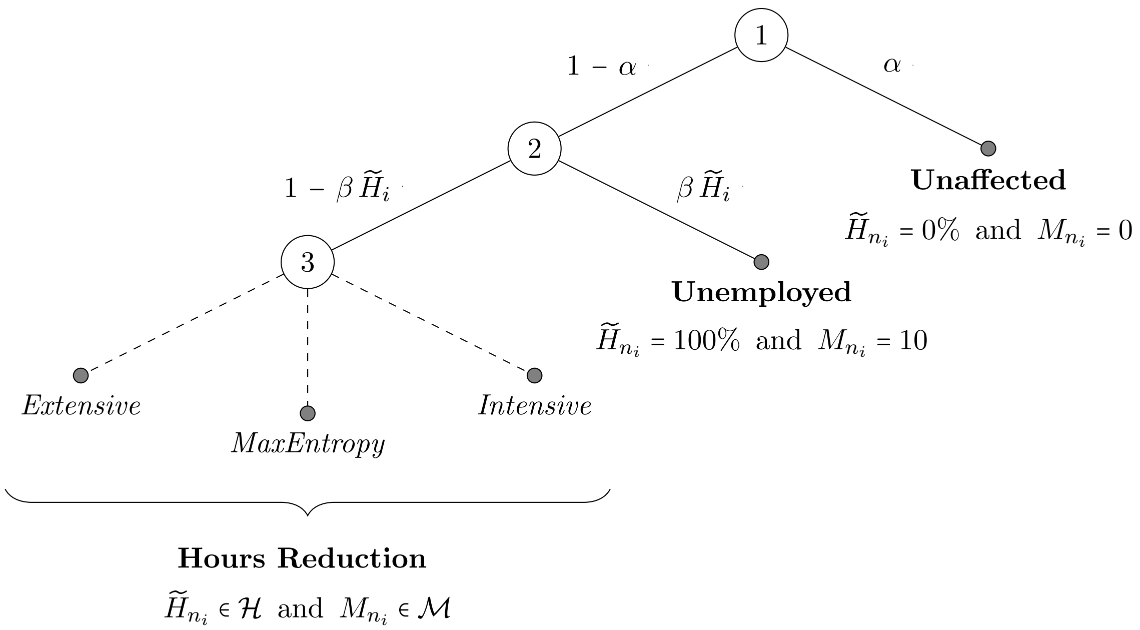

NOTE. — The figure displays the three stages of our "COVID-19 lottery". In the first stage, persons who are potentially affected by the COVID-19 induced output shock draw from a uniform distribution. A person with index n_i in industry-by-input cell i continues working at their pre-crisis working hours with probability α. In this case, their realized relative hours reduction, $\widetilde{H}_{n_i}$, is zero and the number of months they are affected by the crisis, M_{n_i}, is also zero, and the lottery ends (outcome "Unaffected"). Otherwise, the person reaches the second stage of the lottery and takes another draw from a uniform distribution. With probability $\beta\cdot\widetilde{H}_i$ the person loses their job, where $\widetilde{H}_i$ is the average relative loss in working hours in cell i predicted by our labor demand model. With the complementary probability $1-\beta\cdot\widetilde{H}_i$ the person stays employed or self-employed, but suffers a relative loss in working hours (outcome "Hours reduction"). If a person reaches the third stage of the lottery, this relative loss is determined using one of three different scenarios (*MaxEntropy, Extensive, Intensive*; see Section 2.3) for translating the average cell-specific relative hours loss $\widetilde{H}_i$ to realizations for the individual relative losses $\widetilde{H}_{n_i}$ and a realization for the number of months in which the person is affected by this relative hours loss. Depending on the scenario, the realizations of the relative hours loss take one of the values $\widetilde{H}_{n_i}\in\mathcal{H}:=\{0.1,\ 0.2,\ldots,1\}$ and the realizations for the number of months in which this loss occurs takes one of the values $M_{n_i}\in\mathcal{M}:=\{1,\ 2,\ldots,\ 10\}$. For all three scenarios, we ensure that $E\left[\widetilde{H}_{n_i}\right]=(1-\alpha)\cdot\left[\left(1-\beta\widetilde{H}_i\right)\cdot E\left[\widetilde{H}_{n_i}|s\right]+\beta\widetilde{H}_i\cdot 10/12\right]=\widetilde{H}_i$ holds, where $s\in\{MaxEntropy,\ Extensive,\ Intensive\}$, i.e., for each individual belonging to a cell $i=1,\ldots,648$ the expected relative loss in hours over all three stages of the lottery equals the average relative hours reduction in cell i predicted by our labor demand model. Source: Own illustration.
